# Fixed-Time Active Disturbance Rejection Temperature–Pressure Decoupling Control for a High-Flow Air Intake System

**DOI:** 10.3390/e27080880

**Published:** 2025-08-20

**Authors:** Louyue Zhang, Hehong Zhang, Duoqi Shi, Zhihong Dan, Xi Wang, Chao Zhai, Gaoxi Xiao, Zhouzhe Xu

**Affiliations:** 1 School of Energy and Power Engineering, Beihang University, Beijing 100191, China; louyue1225@buaa.edu.cn (L.Z.); xwang@buaa.edu.cn (X.W.); 2 College of Computer and Data Science, Fuzhou University, Fuzhou 350108, China; hzhang030@e.ntu.edu.sg (H.Z.); 18260628082@163.com (Z.X.); 3 Science and Technology on Altitude Simulation Laboratory, Sichuan Gas Turbine Establishment, AECC, Mianyang 621000, China; dzh798318_cym@163.com; 4 School of Automation, China University of Geosciences, Wuhan 430074, China; zhaichao@amss.ac.cn; 5 School of Electrical and Electronic Engineering, Nanyang Technological University, Singapore 639798, Singapore; egxxiao@ntu.edu.sg

**Keywords:** altitude test facility, flight environment simulation system, active disturbance rejection control, fixed-time control, temperature–pressure decoupling control, sliding-mode controller, super-twisting algorithm

## Abstract

High-flow aeroengine transient tests involve strong coupling and external disturbances, which pose significant challenges for intake environment simulation systems (IESSs). This study proposes a compound control scheme that combines fixed-time active disturbance rejection with static decoupling methods. The scheme integrates a fixed-time sliding-mode controller (FT-SMC) and a super-twisting fixed-time extended-state observer (ST-FT-ESO). A decoupling transformation separates pressure and temperature dynamics into two independent loops. The observer estimates system states and total disturbances, including residual coupling, while the controller ensures fixed-time convergence. The method is deployed on a real-time programmable logic controller (PLC) and validated through hardware-in-the-loop (HIL) simulations under representative high-flow scenarios. Compared to conventional linear active disturbance rejection decoupling control (LADRDC), the proposed scheme reduces the absolute integral error (AIE) in pressure and temperature tracking by 71.9% and 77.9%, respectively, and reduces the mean-squared error (MSE) by 46.0% and 41.3%. The settling time improves from over 5 s to under 2 s. These results demonstrate improved tracking accuracy, faster convergence, and enhanced robustness against disturbances.

## 1. Introduction

Altitude simulation testing is an essential method for aeroengine design, performance evaluation, model refinement, technology development, and fault reproduction and mitigation. A key component of this testing is the precise replication of the engine’s in-flight operating conditions. The intake environment simulation system (IESS) is a critical subsystem within the altitude simulation facility [1,2]. Under transient conditions—such as thrust surges, inertial starts, and acceleration phases—the IESS must maintain high dynamic performance. At the same time, it must ensure stable pressure regulation to satisfy strict requirements regarding response speed, overshoot, and steady-state accuracy [3].

For temperature–pressure control in high-flow aeroengine inlet systems, existing research has concentrated on three main areas: (1) *Robust disturbance rejection capability:* During altitude tests, the inlet mass flow rate can vary rapidly—rates may exceed $30\;\mathrm{kg}\;s^{-2}$. The system characteristics change accordingly, demanding control strategies that maintain excellent dynamic response and disturbance suppression over a wide disturbance range. (2) *Multivariable decoupling in multi-actuator structures*: High-flow conditions induce strong coupling between pressure and temperature, and rapid changes in either variable can severely degrade control performance. (3) *Estimation and compensation of internal and external uncertainties*: A high-flow IESS constitutes a high-order, nonlinear, and high-inertia thermo-fluid system, for which establishing an accurate mathematical model is particularly challenging. Internally, it suffers drift in valve-flow characteristics, actuator hysteresis and saturation, sensor noise, and thermal deformation and parameter perturbations in piping. Externally, it endures severe flow disturbances from the engine and environmental influences. The interaction of these uncertainties undermines the effectiveness of model-based advanced controllers [4].

Therefore, an advanced control strategy is urgently needed—one that integrates robustness, efficient multivariable decoupling, and real-time disturbance estimation and rejection. Such a strategy would overcome the limitations imposed by model mismatch, strong coupling, and large disturbances on the transient control performance of a high-flow IESS, meeting the rigorous demands of aeroengine altitude simulation testing.

Common strategies for multivariable control include decentralised control, centralised control, and decoupling control [5,6]. Decentralised methods treat inter-channel coupling as disturbances [7], but they often fail when coupling is strong. Centralised approaches—such as internal-model control [8,9], predictive control [10], robust control [11,12], and adaptive control [13]—can handle system uncertainties but require complex models and heavy computation. Adaptive schemes in particular demand significant time for online parameter tuning, making them unsuitable for rapid transients. In contrast, decoupling control separates the IESS into independent loops, greatly reducing coupling effects on regulation quality and allowing each loop’s controller to be designed independently. Active disturbance rejection decoupling control (ADRDC) treats the coupling as a disturbance between loops, estimates it, and compensates for it to achieve high performance.

Extensive work has addressed coordinated multi-actuator regulation of inlet pressure and temperature. The Arnold Engineering Development Complex (AEDC) applied process control-based strategies to hypersonic engine pressure and temperature transients [14,15]. Researchers at Stuttgart University designed a feed-forward multivariable controller, achieving substantial improvements in transient regulation [16,17]. Others have used linear ADRC for inlet-pressure control, enhancing overall performance [18]. However, true pressure–temperature decoupling remains in its infancy: most studies are theoretical, with few practical implementations. Lun et al. [19] proposed a linear parameter-varying (LPV)-based decoupling to improve PID performance, but proportional–integral–derivative (PID) control still suffers from large overshoot or divergence under severe disturbances. Zhu et al. [20] applied LPV μ-synthesis to handle large uncertainty, yet this approach demands extensive model linearisation and frequent updates.

Active disturbance rejection control (ADRC), introduced by Han, compensates for multivariable coupling as disturbances via a nonlinear extended-state observer (NESO) [21,22]. To simplify tuning, Gao et al. developed linear ADRC (LADRC) [23,24]. More recently, fixed-time stability theory has been incorporated into ADRC: a fixed-time ESO in a voltage regulator achieved faster convergence and higher estimation accuracy than its conventional counterpart [25]. A missile flight controller used a fixed-time ESO to precisely estimate unknown acceleration disturbances [26]. In wheeled mobile robots, a fixed-time sliding-mode controller paired with a novel fixed-time ESO delivered global fixed-time stability and superior dynamic and steady-state performance [27]. The fixed-time sliding-mode controller offers fast convergence with a guaranteed upper bound on settling time, regardless of initial conditions, while remaining suitable for real-time implementation. Combined with a fixed-time ESO, it reduces noise and further boosts disturbance rejection, guaranteeing stabilisation within a fixed time [26,27].

Recent studies show that pairing a fixed-time sliding-mode controller with a fixed-time ESO delivers efficient, high-quality control in uncertain, strongly coupled systems that use multiple actuators. High-flow IESSs need fast and stable pressure–temperature regulation during rapid transients and severe disturbances. Transient tests add further challenges: sharp flow changes, strong multivariable coupling, multi-source noise, and tight settling time limits. To meet these challenges, this paper embeds the super-twisting algorithm into a fixed-time ESO, develops a super-twisting fixed-time ESO (ST-FT-ESO), and combines it with a fixed-time sliding-mode controller to form a fixed-time active disturbance rejection compound decoupling controller (FT-ADRCDC) for high-flow IESS.

The main contributions of this article are listed as follows:

1. We introduce a static decoupling method to remove the IESS’s static coupling. By defining virtual control inputs, the system is split into pressure and temperature loops, each equipped with a dedicated fixed-time sliding-mode controller (FT-SMC) and super-twisting fixed-time ESO (ST-FT-ESO) to achieve high-quality decoupling under high airflow.
2. We employ ST-FT-ESO for rapid and precise estimation of dynamic coupling and other disturbances and FT-SMC as the core controller to reject the total disturbance. Lyapunov analysis shows that the ST-FT-ESO converges in a fixed time and, together with the FT-SMC law, guarantees fixed-time stability of the entire closed-loop system.
3. We implement FT-ADRCDC on a hardware-in-the-loop (HIL) simulation platform for the IESS and compare it with LADRC, demonstrating faster response and superior stability in rapid transient tests.

The remainder of this article is organised as follows: Section 2 introduces the high-flow intake environment simulation system. Section 3 presents the pressure and temperature decoupling design of the IESS. Section 4 proposes the active disturbance rejection compound decoupling controller. Section 5 analyses system stability via Lyapunov methods. Section 6 reports experimental simulation and validation results. Section 7 concludes the paper.

## 2. Intake Environment Simulation System

**Figure 1:**
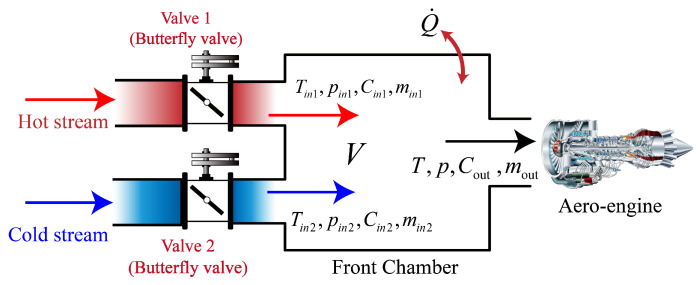
Simplified diagram of the IESS.

The structure and basic operational process of the IESS are illustrated in Figure 1. First, real-time pressure and temperature signals are acquired by dedicated sensors within the system. A feedback controller is then employed to generate opening commands for two control valves, which regulate the motion of valve 1 and valve 2. These valves adjust the respective flows of hot and cold air streams into the front-chamber cavity, thereby enabling precise closed-loop control of both pressure and temperature within the cavity.

The controlled variables—intake pressure and temperature—are influenced by several component dynamics, including the flow characteristics of the front-chamber cavity, the actuation and flow behaviour of the control valves, and the airflow dynamics associated with the tested aeroengine. The detailed modelling process is described in [4]. For completeness, the key modelling results are summarised below.

### 2.1. Front-Chamber Cavity Model

The front-chamber cavity contains two air intakes and one exhaust. The first air intake is a high-temperature airflow with temperature $T_{\mathrm{in}1}$, and the second intake is a low-temperature airflow with temperature $T_{\mathrm{in}2}$, controlled by Control Valve 1 and Control Valve 2, respectively. Because the main duct of the high-flow intake system has a large diameter (>1.5 m) and a relatively low gas velocity (<100 m s^−1^), the flow inside the duct can be reasonably assumed to be perfectly mixed [4,28]. Thus the dynamic characteristics of the intake pressure and temperature are

(1) $$\begin{array}{cc} \frac{dp}{dt} & =\frac{R}{V}\left[\left(T-\frac{h-RT}{c_{p}-R}\right)\left({\dot{m}}_{\mathrm{in}1}+{\dot{m}}_{\mathrm{in}2}-{\dot{m}}_{\mathrm{out}}\right)\right]\\ & \;+\frac{R\dot{Q}}{V(c_{p}-R)}+\frac{R}{V(c_{p}-R)}\left[\left(h_{\mathrm{in}1}+\frac{C_{\mathrm{in}1}^{2}}{2}\right){\dot{m}}_{\mathrm{in}1}+\left(h_{\mathrm{in}2}+\frac{C_{\mathrm{in}2}^{2}}{2}\right){\dot{m}}_{\mathrm{in}2}-\left(h+\frac{C_{\mathrm{out}}^{2}}{2}\right){\dot{m}}_{\mathrm{out}}\right], \end{array}$$

(2) $$\begin{array}{cc} \frac{dT}{dt} & =\frac{RT}{pV(c_{p}-R)}\left[-\left({\dot{m}}_{\mathrm{in}1}+{\dot{m}}_{\mathrm{in}2}-{\dot{m}}_{\mathrm{out}}\right)\left(h-RT\right)+\dot{Q}\right]\\ & \;+\frac{RT}{pV(c_{p}-R)}\left[\left(h_{\mathrm{in}1}+\frac{C_{\mathrm{in}1}^{2}}{2}\right){\dot{m}}_{\mathrm{in}1}+\left(h_{\mathrm{in}2}+\frac{C_{\mathrm{in}2}^{2}}{2}\right){\dot{m}}_{\mathrm{in}2}-\left(h+\frac{C_{\mathrm{out}}^{2}}{2}\right){\dot{m}}_{\mathrm{out}}\right]. \end{array}$$

where $p,T,V,c_{p},h,C_{\mathrm{out}}$ and ${\dot{m}}_{\mathrm{out}}$ denote cavity pressure, temperature, volume, specific heat at constant pressure, enthalpy, average outflow velocity and mass flow rate, respectively; ${\dot{m}}_{\mathrm{in}1},h_{\mathrm{in}1},$ and $C_{\mathrm{in}1}$ and ${\dot{m}}_{\mathrm{in}2},h_{\mathrm{in}2},$ and $C_{\mathrm{in}2}$ are analogous quantities for the hot- and cold-air paths; *R* is the gas constant; and $\dot{Q}$ is the convective heat transfer between the cavity and its surroundings.

Throughout this work, the specific heat at constant pressure, $c_{p}$, and the enthalpy, *h*, of dry air are treated as temperature-dependent. For 199–2201 K, we use the NASA CEA eight-coefficient polynomial [29]

$$\frac{c_{p}\left(T\right)}{R}=\sum_{i=0}^{8}A_{i}{\left(\frac{T}{1000}\right)}^{i},\;\frac{h(T)}{RT}=\sum_{i=0}^{8}\frac{A_{i}}{i+1}\;{\left(\frac{T}{1000}\right)}^{i+1},$$

and the complete coefficient list is given in Appendix A.

### 2.2. Motion and Flow Characteristics of the Control Valve

The valve actuator is modelled by a first-order inertial element identified through system identification:

$$\chi_{a}\left(s\right)=\frac{K_{a}}{t_{a}s+1}\;u\left(s\right),$$

$$\dot{m}=\varphi A_{0}\chi_{a}\sqrt{2\rho p_{1}},$$

where $K_{a}$ is the equivalent gain, $t_{a}$ is the time constant (for the valve shown in Figure 1, $t_{a}=1.6$), $\chi_{a}$ denotes the valve displacement, *u* is the control input, φ is the flow coefficient, $A_{0}$ is the flow area, ρ is the air density, and $p_{1}$ is the upstream pressure.

In practical operating conditions, mechanical backlash and clearances between the valve plate and linkage structures introduce a degree of hysteresis into the actuator’s response. This effect can be equivalently represented by an input delay term $e^{-\theta s}$, which is incorporated into the simulation system (with θ=0.15). From the controller’s perspective, such delay is difficult to observe and is treated as an external disturbance.

### 2.3. Airflow Model of the Aeroengine

The IESS supplies conditioned air to the aeroengine. During transient tests, engine flow variations constitute the largest disturbance source. A data-driven approximation is

$${\dot{W}}_{\mathrm{engine}}=f_{e}\left(H,\;\mathrm{Ma},\;A_{\mathrm{pla}}\right),$$

where *H* is the flight altitude, Ma the flight Mach number, and $A_{\mathrm{pla}}$ is the throttle-lever angle. Strong nonlinearity under different operating conditions leads to significant model uncertainty; the above function serves only as an approximation.

## 3. Pressure and Temperature Decoupling Design of the IESS

To eliminate the static coupling part of the system control parameters, virtual control parameters are introduced into the system for the decoupling design. The system output and input are defined as ${\left[\begin{array}{cc} p & T \end{array}\right]}^{T}$ and ${\left[\begin{array}{cc} u_{1} & u_{2} \end{array}\right]}^{T}$, respectively, where *p* and *T* are the intake pressure and temperature in the front-chamber cavity, and $u_{1}$ and $u_{2}$ refer to the control quantities of Control Valve 1 and Control Valve 2.

### 3.1. Decoupling Design

#### 3.1.1. Affine Model of the IESS

According to Equations (4) and (5), the flow characteristic equations of control valves, as well as the outflow equation of the front-chamber cavity, can be described as

(3) $$\left\{\begin{array}{c} {\dot{m}}_{in1}=\varphi A_{0}\sqrt{2\rho_{1}p_{1,1}}\chi_{a,1}\\ {\dot{m}}_{in2}=\varphi A_{0}\sqrt{2\rho_{2}p_{1,2}}\chi_{a,2} \end{array}\right.$$

(4) $${\dot{m}}_{out}=Wa_{engine}=f_{e}\left(H,Ma,A_{pla}\right)$$

Therein, $p_{1,1}$ is the upstream pressure of Control Valve 1, and $p_{1,2}$ is the upstream pressure of Control Valve 2.

Combining Equations (1), (2), (3) and (4), the nonlinear affine model of the system is given as

(5) $$\left\{\begin{array}{c} \dot{p}=f_{p}^{*}\left(p,T\right)+b_{11}^{*}\chi_{a,1}+b_{12}^{*}\chi_{a,2}\\ \dot{T}=f_{T}^{*}\left(T,p\right)+b_{21}^{*}\chi_{a,1}+b_{22}^{*}\chi_{a,2} \end{array}\right.$$

where $f_{p}^{*}\left(p,T\right)$ and $f_{T}^{*}\left(T,p\right)$ are the total disturbances of the intake pressure and temperature loop in system (8), which contains system model error, dynamic coupling, uncertainties, etc. $b_{11}^{*},b_{12}^{*},b_{21}^{*},$ and $b_{22}^{*}$ are the real-time opening coefficients of the valves, detailed as follows:

$$\begin{array}{cc} b_{11}^{*}= & \left[\frac{R}{V}\left(T-\frac{\left(h-RT\right)}{\left(c_{p}-R\right)}\right)+\frac{R}{V\left(c_{p}-R\right)}\left(h_{in1}\;+\frac{C_{in1}^{2}}{2}\;\right)\right]\\ \; & \varphi A_{0}\sqrt{2\rho_{1}p_{1,1}} \end{array}$$

$$\begin{array}{cc} b_{12}^{*}= & \left[\frac{R}{V}\left(T-\frac{\left(h-RT\right)}{\left(c_{p}-R\right)}\right)+\frac{R}{V\left(c_{p}-R\right)}\left(h_{in2}\;+\frac{C_{in2}^{2}}{2}\;\right)\right]\\ \; & \varphi A_{0}\sqrt{2\rho_{2}p_{1,2}} \end{array}$$

$$\begin{array}{cc} b_{21}^{*}= & \left[-\frac{RT}{pV\left(c_{p}-R\right)}\left(h-RT\right)+\frac{RT}{pV\left(c_{p}-R\right)}\left(h_{in1}\;+\frac{C_{in1}^{2}}{2}\;\right)\right]\\ \; & \varphi A_{0}\sqrt{2\rho_{1}p_{1,1}} \end{array}$$

$$\begin{array}{cc} b_{22}^{*}= & \left[-\frac{RT}{pV\left(c_{p}-R\right)}\left(h-RT\right)+\frac{RT}{pV\left(c_{p}-R\right)}\left(h_{in2}+\frac{C_{in2}^{2}}{2}\right)\right]\\ \; & \varphi A_{0}\sqrt{2\rho_{2}p_{1,2}} \end{array}$$

#### 3.1.2. Decoupling Design of Valve Control Quantity and System Output

Firstly, the derivative of $V_{a,1}$ and $V_{a,2}$ can be deduced from Equation (3):

(6) $$\left[\begin{array}{c} {\dot{\chi }}_{a,1}\\ {\dot{\chi }}_{a,2} \end{array}\right]=\left[\begin{array}{c} -\frac{1}{t_{a,1}}\;0\\ \;0\;-\frac{1}{t_{a,2}} \end{array}\right]\left[\begin{array}{c} \chi_{a,1}\\ \chi_{a,2} \end{array}\right]+\left[\begin{array}{c} \frac{K_{a,1}}{t_{a,1}}\;0\\ \;0\;\frac{t_{a,2}}{T_{a,2}} \end{array}\right]\left[\begin{array}{c} u_{1}\\ u_{2} \end{array}\right]$$

where $K_{a,1},K_{a,2}$, and $t_{a,1},t_{a,2}$ are the equivalent coefficients and time constants of the control valves, respectively.

Then, the affine nonlinear model of the control parameters and the system outputs is established, as shown below:

(7) $$\left\{\begin{array}{c} \ddot{p}=f_{p}\left(p,T\right)+b_{11}u_{1}+b_{12}u_{2}\\ \ddot{T}=f_{T}\left(T,p\right)+b_{21}u_{1}+b_{22}u_{2} \end{array}\right.$$

where $f_{p}\left(p,T\right)$ and $f_{T}\left(T,p\right)$ are the dynamic coupling part and other unknown disturbances (including system model error, uncertainty, etc.) of the system. $b_{11},b_{12},b_{21},$ and $b_{22}$ are the control coefficients of the valves.

$$\begin{array}{c} f_{p}\left(p,T\right)={\dot{f}}_{p}^{*}\left(p,T\right)+{\dot{b}}_{11}^{*}\chi_{a,1}+{\dot{b}}_{12}^{*}\chi_{a,2}-\frac{b_{11}^{*}}{t_{a,1}}\chi_{a,1}-\frac{b_{12}^{*}}{t_{a,2}}\chi_{a,2} \end{array}$$

$$\begin{array}{c} f_{T}\left(T,p\right)={\dot{f}}_{T}^{*}\left(T,p\right)+{\dot{b}}_{21}^{*}\chi_{a,1}+{\dot{b}}_{22}^{*}\chi_{a,2}-\frac{b_{21}^{*}}{t_{a,1}}\chi_{a,1}-\frac{b_{22}^{*}}{t_{a,2}}\chi_{a,2} \end{array}$$

$$\begin{array}{cc} b_{11} & =\frac{b_{11}^{*}K_{a,1}}{t_{a,1}}=[\frac{R}{V}\left(T-\frac{\left(h-RT\right)}{\left(c_{p}-R\right)}\right)\\ \; & +\frac{R}{V\left(c_{p}-R\right)}\left(h_{in1}\;+\frac{C_{in1}^{2}}{2}\;\right)]\varphi A_{0}\sqrt{2\rho_{1}p_{1,1}}\frac{K_{a,1}}{t_{a,1}} \end{array}$$

$$\begin{array}{cc} b_{12} & =\frac{b_{12}^{*}K_{a,2}}{t_{a,2}}=[\frac{R}{V}\left(T-\frac{\left(h-RT\right)}{\left(c_{p}-R\right)}\right)\\ \; & +\frac{R}{V\left(c_{p}-R\right)}\left(h_{in2}\;+\frac{C_{in2}^{2}}{2}\;\right)]\varphi A_{0}\sqrt{2\rho_{2}p_{1,2}}\frac{K_{a,2}}{t_{a,2}} \end{array}$$

$$\begin{array}{cc} b_{21} & =\frac{b_{21}^{*}K_{a,1}}{t_{a,1}}=[-\frac{RT}{pV\left(c_{p}-R\right)}\left(h-RT\right)\\ \; & +\frac{RT}{pV\left(c_{p}-R\right)}\left(h_{in1}\;+\frac{C_{in1}^{2}}{2}\;\right)]\varphi A_{0}\sqrt{2\rho_{1}p_{1,1}}\frac{K_{a,1}}{t_{a,1}} \end{array}$$

$$\begin{array}{cc} b_{22} & =\frac{b_{22}^{*}K_{a,2}}{t_{a,2}}=[-\frac{RT}{pV\left(c_{p}-R\right)}\left(h-RT\right)\\ \; & +\frac{RT}{pV\left(c_{p}-R\right)}\left(h_{in2}+\frac{C_{in2}^{2}}{2}\right)]\varphi A_{0}\sqrt{2\rho_{2}p_{1,2}}\frac{K_{a,2}}{t_{a,2}} \end{array}$$

To simplify the calculation, the virtual control parameters, total disturbances (including the system model error, the dynamic coupling part excluding the control parameters of the system, uncertainty, etc.), and static coupling matrix are defined as U,F, and *B*, respectively.

$$U=\left[\begin{array}{c} U_{1}\\ U_{2} \end{array}\right]=\left[\begin{array}{cc} b_{11} & b_{12}\\ b_{21} & b_{22} \end{array}\right]\left[\begin{array}{c} u_{1}\\ u_{2} \end{array}\right],\;B=\left[\begin{array}{cc} b_{11} & b_{12}\\ b_{21} & b_{22} \end{array}\right]$$

$$F=\left[\begin{array}{c} f_{p}\left(p,T\right)\\ f_{T}\left(T,p\right) \end{array}\right]=\left[\begin{array}{c} F_{1}\\ F_{2} \end{array}\right]$$

Finally, Equation (10) is organised into the following form:

(8) $$\begin{array}{c} \left\{\begin{array}{c} \ddot{p}=F_{1}+U_{1}\\ \ddot{T}=F_{2}+U_{2} \end{array}\right. \end{array}$$

where the static coupling part of the system’s control parameters has been eliminated.

**Figure 2:**
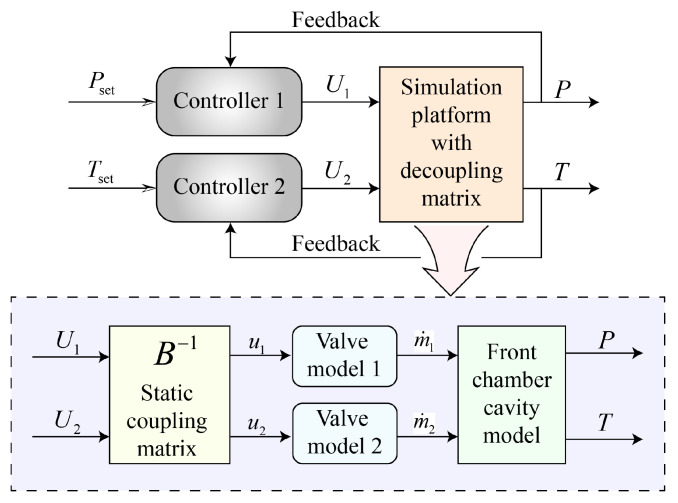
Schematic of the pressure–temperature decoupling control framework.

Figure 2 illustrates the control framework derived above, showing how the virtual inputs $U_{1}$ and $U_{2}$, produced by two independent single-variable controllers, are mapped through the static decoupling matrix $B^{-1}$ to the physical valve commands $u_{1}$ and $u_{2}$. These commands drive the hot- and cold-stream valves, whose mass flow rates, ${\dot{m}}_{1}$ and ${\dot{m}}_{2}$, feed the front-chamber cavity model, generating the controlled pressure *P* and temperature *T* that close the feedback loop.

### 3.2. Static Coupling Matrix Reversibility Analysis

As demonstrated in Section 3.1, the introduction of virtual control variables enables decoupling between the controlled output and these virtual variables. During the practical implementation of the control process, the virtual control variables presented in Equation (11) need to be converted into the actual control parameters by $B^{-1}$. Consequently, it is crucial to evaluate the invertibility of the matrix *B*.

To facilitate the calculation, let the matrices $A=\left[\begin{array}{cc} a_{1} & a_{2}\\ a_{3} & a_{4} \end{array}\right]$ and $C=\left[\begin{array}{cc} c_{1} & 0\\ 0 & c_{4} \end{array}\right]$.

$$\begin{array}{cc} a_{1} & =\frac{R}{V}\left(T-\frac{\left(h-RT\right)}{\left(c_{p}-R\right)}\right)\\ a_{2} & =\frac{R}{V\left(c_{p}-R\right)}\left(h_{in1}\;+\frac{C_{in1}^{2}}{2}\;\right)\\ a_{3} & =-\frac{RT}{pV\left(c_{p}-R\right)}\left(h-RT\right)\\ a_{4} & =\frac{R}{V\left(c_{p}-R\right)}\left(h_{in2}\;+\frac{C_{in2}^{2}}{2}\;\right)\\ c_{1} & =\varphi A_{0}\sqrt{2\rho_{1}p_{1,1}}\frac{K_{a,1}}{t_{a,1}}\\ c_{4} & =\varphi A_{0}\sqrt{2\rho_{2}p_{1,2}}\frac{K_{a,2}}{t_{a,2}} \end{array}$$

The matrix $B=\left[\begin{array}{cc} a_{1}+a_{2} & a_{1}+a_{4}\\ a_{3}+\frac{T}{p}a_{2} & a_{3}+\frac{T}{p}a_{4} \end{array}\right]C$ is obtained; taking the determinants on both sides, it follows that

(9) $$\begin{array}{c} \left|B\right|=\left|\begin{array}{cc} a_{1}+a_{2} & a_{1}+a_{4}\\ a_{3}+\frac{T}{p}a_{2} & a_{3}+\frac{T}{p}a_{4} \end{array}\right|\left|C\right| \end{array}$$

(10) $$\begin{array}{cc} \left|\begin{array}{cc} a_{1}+a_{2} & a_{1}+a_{4}\\ a_{3}+\frac{T}{p}a_{2} & a_{3}+\frac{T}{p}a_{4} \end{array}\right|= & \frac{R^{2}T^{2}}{pV^{2}}\frac{1}{\left(c_{p}-R\right)}\left(h_{in2}-\;h_{in1}+\frac{C_{in2}^{2}}{2}-\frac{C_{in1}^{2}}{2}\right) \end{array}$$

(11) $$\begin{array}{c} \left|C\right|=2\varphi^{2}A_{0}^{2}\sqrt{\rho_{1}\rho_{2}p_{1,1}p_{1,2}}\frac{K_{a,1}}{t_{a,1}}\frac{K_{a,2}}{t_{a,2}} \end{array}$$

where $c_{p}-R>0,h_{in2}+\frac{C_{in2}^{2}}{2}\ll h_{in1}+\frac{C_{in1}^{2}}{2},\varphi \geq 0$, $A_{0}\geq 0$, and $\sqrt{\rho_{1}\rho_{2}p_{1,1}p_{1,2}}\frac{K_{a,1}}{t_{a,1}}\frac{K_{a,2}}{t_{a,2}}>0$.

It can be seen from the above that $\varphi A_{0}\geq 0$, and this equation holds if and only if the valves are completely closed, so when the valve output is not 0, we have

$$\begin{array}{cc} \left|B\right|= & \frac{R^{2}T^{2}}{pV^{2}}\frac{1}{\left(c_{p}-R\right)}\left(h_{in2}-\;h_{in1}+\frac{C_{in2}^{2}}{2}-\frac{C_{in1}^{2}}{2}\right)\\ \; & *2\varphi^{2}A_{0}^{2}\sqrt{\rho_{1}\rho_{2}p_{1,1}p_{1,2}}\frac{K_{a,1}}{t_{a,1}}\frac{K_{a,2}}{t_{a,2}}\neq 0 \end{array}$$

The matrix *B* is invertible only when the valve output is not zero. During the decoupling control process of the system, control valves will always maintain a certain opening; that is, the valve output will not be zero.

**Remark** **1.** *The flight mission profile includes inertial start, descending or climbing at constant Mach number, thrust transient, and so on. During the test process, the inverse matrix of B does not have excessive values for some elements.*

## 4. Design of Fixed-Time Active Disturbance Rejection Decoupling Controller

### 4.1. Pressure and Temperature Decoupling Control Structure

**Figure 3:**
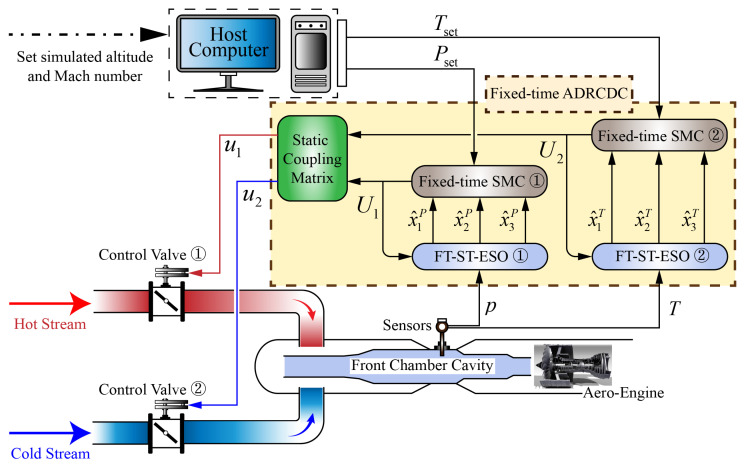
Pressure and temperature decoupling control structure.

A static decoupling strategy is introduced into the intake environment simulation system (IESS) to achieve a one-to-one correspondence between control inputs and environmental parameters. The resulting decoupling control structure is illustrated in Figure 3. Based on this framework, a fixed-time sliding-mode controller (FT-SMC) and a super-twisting fixed-time extended-state observer (ST-FT-ESO) are designed for the pressure and temperature control loops, respectively. The ST-FT-ESO enables rapid and accurate estimation of both system states and the total disturbances for each loop, which are subsequently compensated for within the FT-SMC. This design effectively mitigates complex and time-varying disturbances, ensuring fast and stable system performance. In particular, by accurately estimating the total disturbances—including the dynamic coupling between intake pressure and temperature—the ST-FT-ESO facilitates full decoupling of the controlled variables.

### 4.2. Design of ST-FT-ESO

To achieve complete system decoupling while suppressing measurement noise, we propose a fixed-time extended-state observer enhanced with the super-twisting algorithm. The state definitions of the intake pressure and intake temperature loops remain

$$x_{1}^{p}=p-p_{\mathrm{set}},\;{\dot{x}}_{1}^{p}=x_{2}^{p},\;x_{1}^{\mathrm{Te}}=T-T_{\mathrm{set}},\;{\dot{x}}_{1}^{\mathrm{Te}}=x_{2}^{\mathrm{Te}},$$

leading to the second-order lumped plant models:

(12) $$\left\{\begin{array}{cc} {\dot{x}}_{1}^{p} & =x_{2}^{p},\\ {\dot{x}}_{2}^{p} & =b^{p}U_{1}+d^{p}, \end{array}\right.\;\left\{\begin{array}{cc} {\dot{x}}_{1}^{\mathrm{Te}} & =x_{2}^{\mathrm{Te}},\\ {\dot{x}}_{2}^{\mathrm{Te}} & =b^{\mathrm{Te}}U_{2}+d^{\mathrm{Te}}. \end{array}\right.$$

Assuming that the disturbances $d^{p}$ and $d^{\mathrm{Te}}$ are bounded but unknown, they are promoted to new states:

$$x_{3}^{p}=d^{p},\;x_{3}^{\mathrm{Te}}=d^{\mathrm{Te}},$$

thus yielding third-order plant models:

(13) $$\left\{\begin{array}{cc} {\dot{x}}_{1}^{p} & =x_{2}^{p},\\ {\dot{x}}_{2}^{p} & =b^{p}U_{1}+x_{3}^{p},\\ {\dot{x}}_{3}^{p} & ={\dot{d}}^{p}, \end{array}\right.\;\left\{\begin{array}{cc} {\dot{x}}_{1}^{\mathrm{Te}} & =x_{2}^{\mathrm{Te}},\\ {\dot{x}}_{2}^{\mathrm{Te}} & =b^{\mathrm{Te}}U_{2}+x_{3}^{\mathrm{Te}},\\ {\dot{x}}_{3}^{\mathrm{Te}} & ={\dot{d}}^{\mathrm{Te}}. \end{array}\right.$$

The lumped terms $d^{p}\left(t\right)$ and $d^{\mathrm{Te}}\left(t\right)$ collect all bounded uncertainties, including sensor noise, actuator hysteresis, unmodelled wall heat transfer, minor losses, and other external disturbances. By promoting $d^{\left(\cdot \right)}$ to the extended state $x_{3}^{\left(\cdot \right)}$, the proposed ST-FT-ESO estimates and compensates for these effects online, providing robustness to the control system.

**Figure 4:**
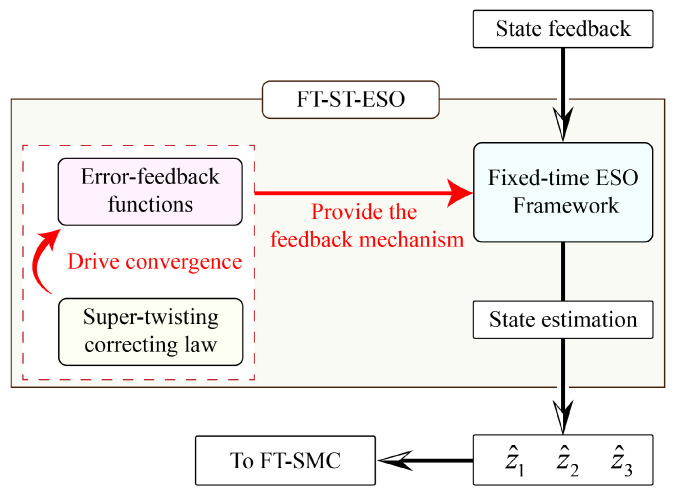
Structure of the ST-FT-ESO framework.

To clarify the interaction between internal components, the modular structure of ST-FT-ESO is illustrated in Figure 4. As shown, the ST-FT-ESO augments the fixed-time observer structure by incorporating a super-twisting correction mechanism into its nonlinear feedback law. The super-twisting algorithm introduces a sign-integrating internal state, *w*, to attenuate high-frequency noise, and its output is modulated by nonlinear gain functions to form the composite feedback terms. These terms are then applied within the fixed-time ESO to ensure finite-time convergence of the estimated states ${\hat{x}}_{i}^{p}$ and ${\hat{x}}_{i}^{\mathrm{Te}}$, even in the presence of bounded disturbances.

Super-twisting correcting law:

For any scalar error *x*, super-twisting feedback is introduced:

$$u_{\mathrm{ST}}\left(x,w\right)=k_{1}{\left|x\right|}^{\rho }\mathrm{sign}\left(x\right)+k_{2}w,\;\dot{w}=\mathrm{sign}\left(x\right),$$

where 0<ρ<1 and $k_{1},k_{2}>0$ are design gains. The auxiliary state *w* integrates the sign function and thus attenuates high-frequency noise.

Error feedback functions:

By combining the super-twisting feedback $u_{\mathrm{ST}}$ with the feedback function of the fixed-time ESO, the pressure-channel ESO feedback function is obtained as follows:

(14) $$\left\{\begin{array}{cc} \phi_{1}^{p}\left(x,w\right) & ={\left|x\right|}^{\beta_{1}^{p}}u_{\mathrm{ST}}\left(x,w\right)+{\left|x\right|}^{\beta_{2}^{p}}u_{\mathrm{ST}}\left(x,w\right),\\ \phi_{2}^{p}\left(x,w\right) & ={\left|x\right|}^{2\beta_{1}^{p}-1}u_{\mathrm{ST}}\left(x,w\right)+{\left|x\right|}^{2\beta_{2}^{p}-1}u_{\mathrm{ST}}\left(x,w\right),\\ \phi_{3}^{p}\left(x,w\right) & ={\left|x\right|}^{3\beta_{1}^{p}-2}u_{\mathrm{ST}}\left(x,w\right)+{\left|x\right|}^{3\beta_{2}^{p}-2}u_{\mathrm{ST}}\left(x,w\right), \end{array}\right.$$

and analogously this holds for $\phi_{j}^{\mathrm{Te}}\left(x,w\right)$ with parameters $\beta_{1}^{\mathrm{Te}}$ and $\beta_{2}^{\mathrm{Te}}$. The exponents satisfy $0<\beta_{1}^{\left(\cdot \right)}<\beta_{2}^{\left(\cdot \right)}<1\;$ and $\rho <\beta_{1}^{\left(\cdot \right)}.$

The FT-ST-ESO dynamics (pressure loop) are as follows:

(15) $$\left\{\begin{array}{cc} e_{1}^{p} & =x_{1}^{p}-z_{1}^{p},\\ {\dot{z}}_{1}^{p} & =z_{2}^{p}+\theta^{p}l_{1}^{p}\;\phi_{1}^{p}\;\left(\frac{e_{1}^{p}}{{\left(\theta^{p}\right)}^{2}},w^{p}\right),\\ {\dot{z}}_{2}^{p} & =b^{p}U_{1}+z_{3}^{p}+l_{2}^{p}\;\phi_{2}^{p}\;\left(\frac{e_{1}^{p}}{{\left(\theta^{p}\right)}^{2}},w^{p}\right),\\ {\dot{z}}_{3}^{p} & =\frac{l_{3}^{p}}{\theta^{p}}\;\phi_{3}^{p}\;\left(\frac{e_{1}^{p}}{{\left(\theta^{p}\right)}^{2}},w^{p}\right),\\ {\dot{w}}^{p} & =\mathrm{sign}(e_{1}^{p}),\\ {\hat{x}}_{i}^{p} & =z_{i}^{p}\;\left(i=1,2,3\right). \end{array}\right.$$

The temperature loop dynamics, $z_{1}^{\mathrm{Te}},z_{2}^{\mathrm{Te}},z_{3}^{\mathrm{Te}},$ and $w^{\mathrm{Te}}$, follow exactly the same pattern with superscript Te.

### 4.3. Design of the FT-SMC

The errors in the system are estimated to be ${\hat{x}}_{1}^{p}$ and ${\hat{x}}_{1}^{Te}$, and the differential errors are estimated to be ${\hat{x}}_{2}^{p}$ and ${\hat{x}}_{2}^{Te}$. To enable better rapid performance in practical applications, let ${\dot{\hat{x}}}_{1}^{p}={\hat{x}}_{2}^{p}$ and ${\dot{\hat{x}}}_{1}^{Te}={\hat{x}}_{2}^{Te}$; the fixed-time sliding-mode surfaces of the system are then formulated as follows:

(16) $$\begin{array}{cc} s^{p} & ={\hat{x}}_{2}^{p}+\alpha_{1}^{p}{\left({\hat{x}}_{1}^{p}\right)}^{\frac{1}{2}+\frac{m_{1}^{p}}{2n_{1}^{p}}+\left(\frac{m_{1}^{p}}{2n_{1}^{p}}-\frac{1}{2}\right)\mathrm{sign}\left(\left|{\hat{x}}_{1}^{p}\right|-1\right)}+\gamma_{1}^{p}{\left({\hat{x}}_{1}^{p}\right)}^{\frac{\delta_{1}^{p}}{q_{1}^{p}}} \end{array}$$

(17) $$\begin{array}{cc} s^{Te} & ={\hat{x}}_{2}^{Te}+\alpha_{1}^{Te}{\left({\hat{x}}_{1}^{Te}\right)}^{\frac{1}{2}+\frac{m_{1}^{Te}}{2n_{1}^{Te}}+\left(\frac{m_{1}^{Te}}{2n_{1}^{Te}}-\frac{1}{2}\right)\mathrm{sign}\left(\left|{\hat{x}}_{1}^{Te}\right|-1\right)}+\gamma_{1}^{Te}{\left({\hat{x}}_{1}^{Te}\right)}^{\frac{\delta_{1}^{Te}}{q_{1}^{Te}}} \end{array}$$

According to the above sliding-mode surfaces, the following FT-SMCs are designed:

(18) $$\begin{array}{cc} u_{1} & =-[\alpha_{1}^{p}\left(\frac{1}{2}+\frac{m_{1}^{p}}{2n_{1}^{p}}+\left(\frac{m_{1}^{p}}{2n_{1}^{p}}-\frac{1}{2}\right)\mathrm{sign}\left(\left|{\hat{x}}_{1}^{p}\right|-1\right)\right)\\ \; & \times {\left({\hat{x}}_{1}^{p}\right)}^{\frac{1}{2}+\frac{m_{1}^{p}}{2n_{1}^{p}}+\left(\frac{m_{1}^{p}}{2n_{1}^{p}}-\frac{1}{2}\right)\mathrm{sign}\left(\left|{\hat{x}}_{1}^{p}\right|-1\right)-1}\\ \; & +sat\left(\gamma_{1}^{p}\frac{\delta_{1}^{p}}{q_{1}^{p}}{\left({\hat{x}}_{1}^{p}\right)}^{\frac{\delta_{1}^{p}}{q_{1}^{p}}-1}{\hat{x}}_{2}^{p},h\right)\\ \; & +\alpha_{2}^{p}{\left(s^{p}\right)}^{\frac{1}{2}+\frac{m_{2}^{p}}{2n_{2}^{p}}+\left(\frac{m_{2}^{p}}{2n_{2}^{p}}-\frac{1}{2}\right)\mathrm{sign}\left(\left|s^{p}\right|-1\right)}\\ \; & +\gamma_{2}^{p}{\left(s^{p}\right)}^{\frac{\delta_{2}^{p}}{q_{2}^{p}}}+{\hat{x}}_{3}^{p}]/b^{p} \end{array}$$

(19) $$\begin{array}{cc} u_{2} & =-[\alpha_{1}^{Te}\left(\frac{1}{2}+\frac{m_{1}^{Te}}{2n_{1}^{Te}}+\left(\frac{m_{1}^{Te}}{2n_{1}^{Te}}-\frac{1}{2}\right)\mathrm{sign}\left(\left|{\hat{x}}_{1}^{Te}\right|-1\right)\right)\\ \; & \times {\left({\hat{x}}_{1}^{Te}\right)}^{\frac{1}{2}+\frac{m_{1}^{Te}}{2n_{1}^{Te}}+\left(\frac{m_{1}^{Te}}{2n_{1}^{Te}}-\frac{1}{2}\right)\mathrm{sign}\left(\left|{\hat{x}}_{1}^{Te}\right|-1\right)-1}\\ \; & +sat\left(\gamma_{1}^{T}\frac{\delta_{1}^{Te}}{q_{1}^{Te}}{\left({\hat{x}}_{1}^{Te}\right)}^{\frac{\delta_{1}^{Te}}{q_{1}^{Te}}-1}{\hat{x}}_{2}^{Te},h\right)\\ \; & +\alpha_{2}^{Te}{\left(s^{Te}\right)}^{\frac{1}{2}+\frac{m_{2}^{Te}}{2n_{2}^{Te}}+\left(\frac{m_{2}^{Te}}{2n_{2}^{Te}}-\frac{1}{2}\right)\mathrm{sign}\left(\left|s^{Te}\right|-1\right)}\\ \; & +\gamma_{2}^{Te}{\left(s^{Te}\right)}^{\frac{\delta_{2}^{Te}}{q_{2}^{Te}}}+{\hat{x}}_{3}^{Te}]/b^{Te} \end{array}$$

We take the intake pressure loop as an example, where $\alpha_{1}^{p},\gamma_{1}^{p},\alpha_{1}^{p},$ and $\gamma_{2}^{p}$ are positive constants and the positive odd integers $m_{1}^{p},n_{1}^{p},m_{2}^{p},n_{2}^{p},\delta_{1}^{p},q_{1}^{p},\delta_{2}^{p}$, and $q_{2}^{p}$ satisfy $m_{1}^{p}>n_{1}^{p}$, $m_{2}^{p}>n_{2}^{p}$, $\delta_{1}^{p}<q_{1}^{p}$, and $\delta_{2}^{p}<q_{2}^{p}$, with $(m_{1}^{p}+n_{1}^{p})/2$, $(m_{2}^{p}+n_{2}^{p})/2$, $(\delta_{1}^{p}+q_{1}^{p})/2$, and $(\delta_{2}^{p}+q_{2}^{p})/2$ being positive odd integers. The parameters in another loop adopt the same definition.

The singularity terms ${\left({\hat{x}}_{1}^{p}\right)}^{\frac{\delta_{1}^{p}}{q_{1}^{p}}-1}{\hat{x}}_{2}^{p}$ and ${\left({\hat{x}}_{1}^{T}\right)}^{\frac{\delta_{1}^{Te}}{q_{1}^{Te}}-1}{\hat{x}}_{2}^{Te}$ may cause a singularity problem; a saturation function, sat, is adopted to avoid this situation:

$$sat\left(a,b\right)=\left\{\begin{array}{cc} a, & \left|a\right|<b\\ b\mathrm{sign}\left(a,,\right) & \left|a\right|\geq b \end{array}\right.$$

## 5. System Stability Analysis

This section formulates the formal theorem and supporting lemmas required for the proposed ST-FT-ESO and for the closed-loop control system of the IESS, and, for each system, it provides a rigorous fixed-time stability proof.

### 5.1. Stability of the ST-FT-ESO

(1) Homogeneity property

**Lemma** **1.** *We consider the pressure loop observer dynamics (15) with error state $\xi^{p}={\left[e_{1}^{p},\;e_{2}^{p},\;e_{3}^{p},\;w^{p}\right]}^{\;⊤}.$ We then introduce the dilation*

$$\Delta_{\lambda }:\;\xi \mapsto D_{\lambda }\xi ,\;D_{\lambda }=\mathrm{diag}\left(\lambda^{r_{1}},\lambda^{r_{2}},\lambda^{r_{3}},\lambda^{r_{4}}\right),$$

*with $r_{1}=1/\left(1-\rho \right),\;r_{2}=r_{1}+1,\;r_{3}=r_{1}+2,\;$ and $r_{4}=r_{2}$, 0<ρ<1. Then $f\left(D_{\lambda }\xi \right)=\lambda^{q}D_{\lambda }f\left(\xi \right)$ with q=−ρ/(1−ρ)<0; hence the ST-FT-ESO is homogeneous with a negative degree.*

**Proof.**  Each term of (15) is scaled and its exponent is compared with the corresponding row of $D_{\lambda }$. In particular, $|e_{1}^{p}{|}^{\rho }\mathrm{sign}\left(e_{1}^{p}\right)$ scales as $\lambda^{r_{1}\rho }=\lambda^{q+r_{2}}$ and ${\dot{w}}^{p}=\mathrm{sign}\left(e_{1}^{p}\right)=\lambda^{q+r_{4}}\mathrm{sign}\left(e_{1}^{p}\right)$ because $q+r_{4}=0$. All higher-order terms inherit the same degree. □

(2) Fixed-time convergence of the ESO

**Theorem** **1** (ESO fixed-time stability)**.** *Assume $\left|{\dot{d}}^{p}\left(t\right)\right|\leq D_{\dot{d}}$. Select $l_{j}^{p},k_{1},$ and $k_{2}>0$ so that*

(20) $$k_{2}>D_{\dot{d}}+\varepsilon ,\;\varepsilon >0.$$

*With the Lyapunov function*

$$V^{p}=a_{1}|e_{1}^{p}{|}^{\rho +1}+a_{2}|e_{2}^{p}{|}^{\;1+\rho /2}+a_{3}\left|e_{3}^{p}\right|+a_{4}{\left(w^{p}\right)}^{2},\;a_{i}>0,$$

*one has*

$${\dot{V}}^{p}\leq -{\tilde{c}}_{1}{\left(V^{p}\right)}^{\alpha }-{\tilde{c}}_{2}{\left(V^{p}\right)}^{\beta },\;\alpha =\frac{\rho }{\rho +1}\in \left(0,1\right),\;\beta =1+\frac{\rho }{2}>1,$$

*for some ${\tilde{c}}_{1},{\tilde{c}}_{2}>0$. Consequently*

$$T_{\mathrm{ESO}}^{p}\leq \frac{1}{{\tilde{c}}_{1}\left(1-\alpha \right)}+\frac{1}{{\tilde{c}}_{2}\left(\beta -1\right)}$$

*is an initial condition-independent convergence bound.*

**Proof.**  $V^{p}$ is differentiated along (15). Using Lemma 1 and Young’s inequality, one obtains

$${\dot{V}}^{p}\leq -c_{1}|e_{1}^{p}{|}^{\rho +1+\rho }-c_{2}|e_{2}^{p}{|}^{\;1+\rho }-c_{3}{\left|e_{3}^{p}\right|}^{1+\rho /2}-c_{4}{\left(w^{p}\right)}^{2},$$

with $c_{4}>0$ enforced by (20). Because $\rho +1+\rho =\left(\rho +1\right)\left(1+\frac{\rho }{\rho +1}\right)=\left(\rho +1\right)\alpha^{-1}$ and the equivalent is true for the other exponents, the right-hand side can be written in the required two-power form. Fixed-time convergence then follows directly from reference [30]. □

### 5.2. Stability of the Closed-Loop IESS

Only the pressure channel is analysed; the temperature loop is symmetric. Let $\Xi^{p}={\left[e_{1}^{p},e_{2}^{p},e_{3}^{p},w^{p},{\hat{x}}_{1}^{p},{\hat{x}}_{2}^{p},s^{p}\right]}^{\;⊤},$ with $s^{p}$ defined in (16).

(1) Sliding-surface reachability

**Lemma** **2.** *With positive gains $\alpha_{2}^{p}$ and $\gamma_{2}^{p}$ and odd integers $m_{2}^{p}>n_{2}^{p}$ and $\;\delta_{2}^{p}<q_{2}^{p}$, the inequality ${\dot{V}}_{1}\leq -c_{1}V_{1}^{\mu }-c_{2}V_{1}^{\nu },$ with 0<μ<1<ν, holds for $V_{1}=\frac{1}{2}{\left(s^{p}\right)}^{2}$. Direct integration gives the fixed-time bound*

$$T_{s}^{p}\leq \frac{1}{c_{1}\left(1-\mu \right)}+\frac{1}{c_{2}\left(\nu -1\right)},$$

*which depends only on the chosen gains.*

**Proof.**  Differentiating $V_{1}$ yields ${\dot{V}}_{1}=s^{p}{\dot{s}}^{p}$. Substituting this into (18) and applying the controller inequalities above produce the stated two-power inequality.

Separating variables and integrating over $V_{1}\in \left(0,V_{1}\left(0\right)\right]$ produces the explicit bound

$$\int_{0}^{T_{s}^{p}}\;\;\;dt\;\;\geq \;\;\int_{0}^{V_{1}\left(0\right)}\;\;\frac{dV_{1}}{c_{1}V_{1}^{\mu }+c_{2}V_{1}^{\nu }}\;\;\leq \;\;\frac{V_{1}{\left(0\right)}^{1-\mu }}{c_{1}\left(1-\mu \right)}+\frac{1}{c_{2}\left(\nu -1\right)}.$$

Since $V_{1}\left(0\right)\leq 1$ by construction (surface normalisation), the right-hand side never exceeds the bound in the lemma. □

(2) Vanishing of the position error

**Lemma** **3.** *After the surface $s^{p}=0$ is reached, the reduced dynamics satisfy ${\dot{\hat{x}}}_{1}^{p}=-\alpha_{1}^{p}|{\hat{x}}_{1}^{p}{|}^{\mu }-\gamma_{1}^{p}{\left|{\hat{x}}_{1}^{p}\right|}^{\nu }$, with0<μ<1<ν. Choosing gains so that $m_{1}^{p}>n_{1}^{p},\;\delta_{1}^{p}<q_{1}^{p}$ gives the fixed-time bound*

$$T_{x}^{p}\leq \frac{1}{\alpha_{1}^{p}\left(1-\mu \right)}+\frac{1}{\gamma_{1}^{p}\left(\nu -1\right)}.$$

**Proof.**  Let $V_{2}=\left|{\hat{x}}_{1}^{p}\right|$. Then ${\dot{V}}_{2}\leq -\alpha_{1}^{p}V_{2}^{\mu }-\gamma_{1}^{p}V_{2}^{\nu }$. Integration exactly as in Lemma 2 yields the stated bound. □

(3) Closed-loop fixed-time stability

**Theorem** **2.** *Under the conditions of Theorem 1 and Lemmas 2 and 3, the origin $\Xi^{p}=0$ has global fixed-time stability. All errors vanish for*

(21) $$t\;\geq \;T_{\mathrm{tot}}\;=\;T_{\mathrm{ESO}}^{p}+T_{s}^{p}+T_{x}^{p},$$

*where each component of time is initial condition-independent.*

**Proof.**  (i) The observer errors converge in time $T_{\mathrm{ESO}}^{p}$ (Theorem 1); (ii) once they vanish, the sliding surface is reached in $T_{s}^{p}$ (Lemma 2); and (iii) on $s^{p}=0$, the position error decays in $T_{x}^{p}$ (Lemma 3). From reference [31], the sum of the fixed-time subsystems remains stable in fixed time, giving the stated bound. □

**Remark** **2.** *The saturation operator sat(·,h) reduces ${\hat{x}}_{2}^{p}$ so that $|{\hat{x}}_{1}^{p}{|}^{\frac{\delta_{1}^{p}}{q_{1}^{p}}-1}\mathrm{sat}\left({\hat{x}}_{2}^{p},h\right)\leq h^{\;1-\frac{\delta_{1}^{p}}{q_{1}^{p}}}\;,$ ensuring every derivative in the Lyapunov analysis is finite and the negative definiteness of $\dot{V}$ is preserved.*

## 6. Experimental Simulation and Validation Analysis

In this section, we develop a real-time simulation platform and configure an aeroengine flight mission to validate the proposed FT-ADRCDC algorithm through simulation and assess its performance. For benchmarking, the linear active disturbance rejection decoupling control (LADRDC) method is implemented under identical conditions, allowing for a direct comparison of algorithmic performance.

### 6.1. Simulation Verification Platform Setup

**Figure 5:**
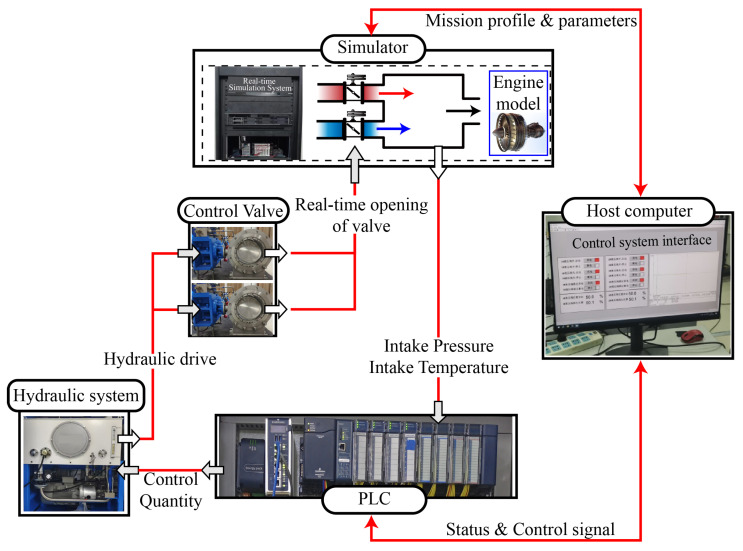
HIL simulation platform.

To verify the feasibility of the FT-ADRCDC in engineering applications, experimental simulations are conducted on a hardware-in-the-loop (HIL) simulation platform of the IESS. As shown in Figure 5, the HIL simulation platform comprises a host computer, a programmable logic controller (PLC), a hydraulic system, two butterfly control valves, and a real-time simulator. The host computer provides the human–machine interface: it sets the controller and model parameters, displays both environmental and intermediate variables, and converts flight setpoints (altitude and Mach number) into temperature- and pressure-based control commands. The simulator deploys the mathematical model of the high-flow intake system, including the valve-flow model, the cavity temperature and pressure models, and the aeroengine model, and replicates the physical input/output environment. The PLC runs the proposed control algorithm, receives feedback from the simulator and commands from the host computer, and outputs control signals in real time. The hydraulic system executes these control signals to drive the butterfly valves. The valves’ openings are measured by displacement sensors, and these signals are sent back to the simulator.

In the HIL simulation tests, the platform reproduces multiple flight conditions encountered in the high-flow altitude test:

Task 1: Constant-altitude ascent/descent and level-flight acceleration.
Task 2: Simultaneous variation in Mach number and altitude.
Task 3: Thrust transient: sudden engine flow change under constant altitude and Mach number.

### 6.2. Real-Time Implementation of Control Algorithm

The ST-FT-ESO and FT-SMC are executed on a GE PACSystems RX3i PLC (CPU CPE330, 1.2 GHz quad-core). All control function blocks are written in IEC 61131-3 Structured-Text and scheduled in a single high-priority task with a 20 ms period (50 Hz). For each scan, the PLC receives intake pressure and temperature from the real-time simulator via EtherCAT, updates the observer by explicit Euler integration, evaluates the sliding-mode control laws, and issues analogue valve commands.

Performance profiling using the PLC’s built-in diagnostics indicates that the complete algorithm consumes less than one third of the available CPU budget, and the end-to-end latency—from the sensor to the actuator—is two orders of magnitude smaller than the dominant cavity time constants. Hardware-in-the-loop tests confirm that the PLC code is numerically equivalent to the offline Simulink model, thereby ensuring that the subsequent simulation results faithfully reflect real-time operational characteristics.

### 6.3. Experimental Task Setup

Task 1: This test evaluates the control system’s simulation performance under level-flight acceleration and constant-Mach-number ascent/descent conditions.

- 0–30 s (level-flight acceleration) at fixed altitude of 8 km:
  – 0–5 s: Mach number, 0.5; throttle, 20°.
  – 5–15 s: uniform acceleration to Mach number of 0.75 (hold to 20 s) and throttle of 22°.
  – 20–28 s: uniform acceleration to Mach number of 0.9 (hold to 30 s) and throttle of 24°.
- 30–75 s (constant-Mach-number ascent/descent) at Mach number of 0.9 and throttle of 24°:
  – 30–38 s: climb from 8 km to 10 km (hold to 43 s).
  – 43–53 s: climb from 10 km to 12 km (hold to 58 s).
  – 58–63 s: descend from 12 km to 11 km.

Task 2: This test evaluates performance under simultaneous variation in Mach number and altitude (75–110 s):

- Altitude: 11 km → 5 km (uniform descent).
- Mach number: 0.9 → 0.5 (uniform deceleration).
- Throttle: 24° → 10° (uniform decrease).

Task 3: This test evaluates the control system’s response to a thrust transient (110–180 s):

- Altitude: hold at 5 km; Mach number: hold at 0.5.
- 110–130 s: hold throttle at 10°.
- 130–135 s: increase throttle to 48°; hold to 155 s.
- 155–160 s: decrease throttle back to 10°; hold to 180 s.

**Figure 6:**
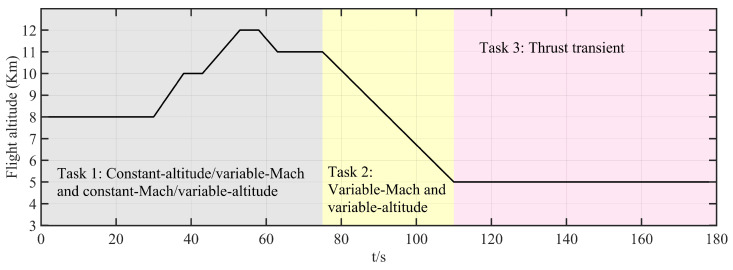
The profile of altitude.

**Figure 7:**
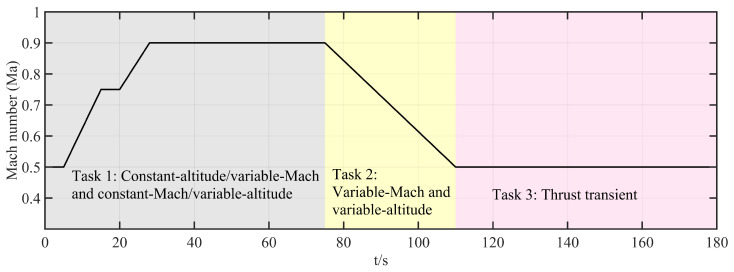
The profile of Mach number.

**Figure 8:**
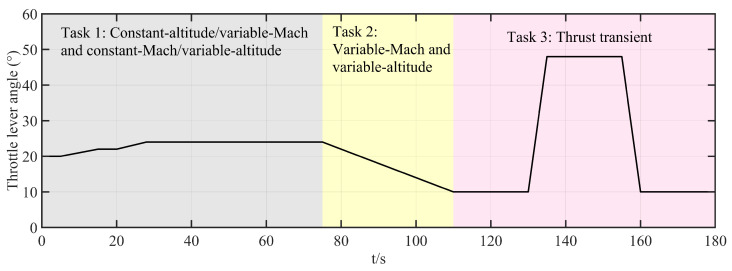
The profile of throttle angle.

**Figure 9:**
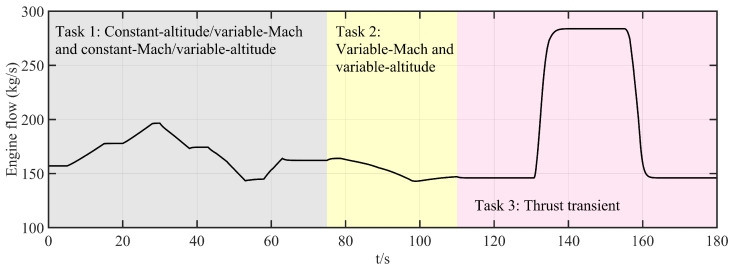
The profile of engine flow.

Tasks 1 and 2 primarily validate the algorithm’s multivariable, multi-actuator decoupling performance. Task 3 focuses on the algorithm’s disturbance rejection capability. The corresponding profiles of altitude, Mach number, throttle angle, and engine flow are shown in Figure 6, Figure 7, Figure 8, and Figure 9, respectively.

### 6.4. Simulation Conditions and Control Parameters

Initial simulation conditions: the hot flow temperature is 283.15 K, the cold flow temperature is 213.15 K, the hot flow pressure is 180 kPa, and the cold flow pressure is 120 kPa. The initial intake temperature is 258.15 K, and the initial intake pressure is 75 kPa. The parameter selection mainly involved configuring a similar bandwidth gain to conduct some comparative experiments. The controller parameters for the control strategies are shown in Table 1. Therein, $Prop_{1},Deri_{1},Prop_{2},$ and $Deri_{2}$ are the parameters of the core controller in LADRDC, and $\omega^{p}$ and $\omega^{Te}$ are the parameters of the ESO in LADRDC.

For the proposed FT-ADRCDC, parameter setting is conducted as follows: The fixed-time ESO component adopts a structured tuning methodology, as summarised in reference [26]. The parameters of the super-twisting algorithm are heuristically tuned based on typical disturbance characteristics observed in large-flow intake systems.

### 6.5. Results Analysis

Using the host computer’s embedded algorithm, the altitude and Mach trajectories shown in Figure 4 and Figure 5 are converted in real time into target front-chamber pressure and temperature references, which are then issued as control commands to the PLC.

**Figure 10:**
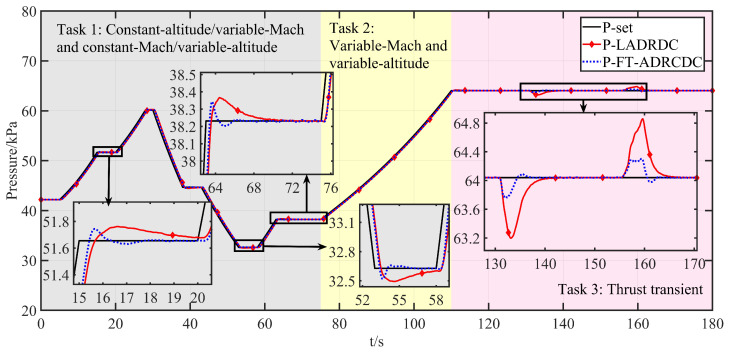
Intake pressure control effect.

**Figure 11:**
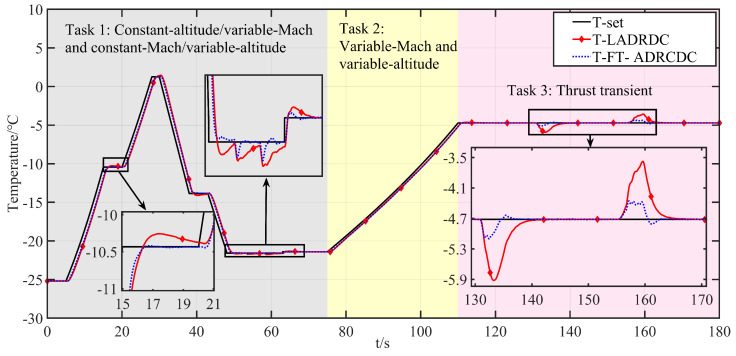
Intake temperature control effect.

**Figure 12:**
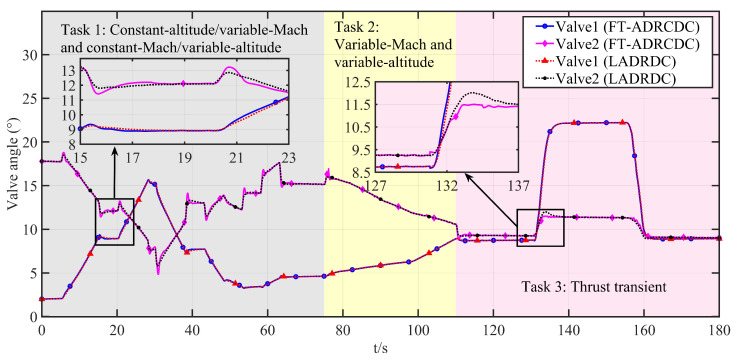
Control valve movement.

The corresponding HIL responses under FT-ADRCDC and LADRDC are illustrated in Figure 10, Figure 11 and Figure 12. As LADRDC is one of the most widely used controllers currently implemented in the facility, it is chosen as the benchmark to ensure practical relevance.

Specifically, Figure 10 and Figure 11 compare the intake pressure and temperature tracking performance of the two controllers. To quantitatively assess the results, three performance indicators are considered: the maximum absolute residual (MAR), the mean-squared error (MSE), and the absolute integral error (AIE). The MAR evaluates the peak instantaneous tracking deviation; the MSE reflects average performance by penalising squared deviations; and the AIE captures the total cumulative deviation over the mission. The computed metrics are summarised in Table 2, showing the consistent advantages of FT-ADRCDC in both pressure and temperature regulation. Figure 12 displays the corresponding actuation trajectories of Control Valve 1 and Control Valve 2. Under the current hardware configuration, all valve movements remain within feasible limits and comply with practical engineering constraints [1].

1. Comparison of Collaborative Decoupling Control Performance for Dual-Variable Temperature–Pressure Tracking

In HIL simulations from 0 to 110 s, the control system was tested on constant-Mach-number ascent/descent, level-flight acceleration, and deceleration–descent tasks. These scenarios validate the algorithm’s ability to perform real-time, cooperative decoupling control of front-chamber temperature and pressure.

As shown in Figure 10 and Figure 11, the proposed FT-ADRCDC delivers superior transient characteristics and faster convergence than LADRDC. Specifically, as shown in Figure 10, under LADRDC, the intake pressure exhibits significant overshoot over the intervals 15–20 s, 53–58 s, and 63–70 s, with convergence times typically exceeding 5 s. In contrast, the FT-ADRCDC ensures pressure convergence within 2 s. Likewise, Figure 11 shows that the intake temperature experiences disturbance due to coupling over 48–53 s, and the FT-ADRCDC controller rejects these disturbances rapidly, yielding markedly improved response quality compared to LADRDC. Table 3 summarises the tracking performance of both algorithms over the 0–110 s interval.

2. Comparison of anti-disturbance abilities under changing flow rates of aeroengine

In Task 3, the throttle lever angle was pushed from 10° to a maximum angle of 48° during the 135–155 s time interval, and the flow rate of the aeroengine changed drastically, which greatly tested the anti-disturbance abilities of the designed algorithm.

During the thrust transient phase under the LADRDC method, the tracking performance over the interval t=110–180 s is summarised in Table 4. As illustrated in Figure 10, Figure 11 and Figure 12, the maximum temperature deviation exceeds 1 °C, while the maximum pressure deviation exceeds 0.8 kPa.

Based on FT-ADRCDC, the absolute integral errors during the 110∼180 s interval are shown in Table 4. As shown in Figure 10, Figure 11 and Figure 12, the maximum temperature deviation in test 1 is approximately 0.5 °C.

Moreover, the maximum pressure deviation is within 1 kPa, at approximately 0.37 kPa. Moreover, the convergence time to reach the reference pressure is significantly faster than that of the LADRDC method.

3. Comparison of convergence rates of FT-ADRDC and LADRDC.

The magnified windows in Figure 10 (pressure loop) and Figure 11 (temperature loop) display every commanded step change or external disturbance applied in Tasks 1–3. Across these transients, the proposed FT–ADRDC trajectory (blue dotted curve) reaches the ±1% pressure band or the ±0.5 °C temperature band within 1–2 s and thereafter remains inside that band, whereas the reference LADRDC scheme (red solid curve) requires 3–8 s to achieve the same settling accuracy under identical conditions.

Using Equation (21) and the gain set of Table 1, the theoretical fixed-time upper bound is $T_{\mathrm{tot}}=2.16\;s$.

All experimentally observed settling times of FT–ADRDC remain below this bound, thereby corroborating the theoretical prediction without further tuning or adjustment.

4. Discussion on algorithmic differences and open issues.

The transient discrepancies between LADRDC and FT-ADRDC in Figure 10, Figure 11 and Figure 12 mainly arise from two structural factors:

(i) Convergence mechanism: FT-ADRDC adopts a fixed-time sliding-mode law whose settling time is upper-bounded and independent of the initial error, whereas the linear feedback in LADRDC converges proportionally to both the initial error and the ESO bandwidth.
(ii) Observer bandwidth: The super-twisting fixed-time ESO (ST-FT-ESO) in FT-ADRDC permits a higher effective bandwidth without noise, enabling disturbance estimation roughly one sampling period earlier and thereby suppressing the temperature spike at *t* = 48–53 s in Figure 11.

Although FT-ADRCDC achieves high tracking accuracy within the current operating window, several limitations warrant closer examination:

(i) The fixed-time gains $\alpha_{i}$, $\gamma_{i}$, and $T_{f}$ are tuned for flows below $500\;\mathrm{kg}\;s^{-1}$; robustness at higher rates remains to be verified. Adaptive scheduling or data-driven tuning merits investigation.
(ii) Actuator saturation and dead-zone effects are currently only handled implicitly, which may degrade performance under extreme throttle commands. A structured anti-windup design could mitigate this issue.
(iii) The current model neglects distributed duct losses and measurement uncertainty; these factors become important in larger, more complex piping networks.

Addressing these aspects would broaden the applicability of the proposed controller and thus represents a worthwhile direction for future investigation.

To sum up, under the condition of disturbance impact where the aeroengine flow changes drastically, the FT-ADRCDC method, equipped with more effective anti-disturbance abilities, can achieve high-quality control of intake pressure and temperature.

## 7. Conclusions

The proposed fixed-time active disturbance rejection compound decoupling controller (FT-ADRCDC) method, which integrates a fixed-time sliding-mode controller (FT-SMC) with a super-twisting fixed-time extended-state observer (ST-FT-ESO), was evaluated through hardware-in-the-loop (HIL) simulations. The following conclusions can be drawn:

1. The algorithm achieves fixed-time convergence by introducing virtual control variables that decouple the intake environment simulation system (IESS) into two single-input, single-output loops. This decoupling mitigates the static coupling between pressure and temperature. The ST-FT-ESO provides rapid, noise-free estimation of the system states and total disturbances. These disturbances are then compensated for in real time by the FT-SMC, enhancing control robustness.
2. Compared with the linear ADRDC (LADRDC) baseline, the proposed FT-ADRCDC achieves significantly better performance across the full simulation window (t=0–180 s). Specifically, the absolute integral error (AIE) for pressure tracking is reduced by 71.9%, and for temperature tracking, it is reduced by 77.9%. The corresponding reductions in the mean-squared error (MSE) are 46.0% and 41.3%, respectively. Moreover, the FT-ADRCDC maintains settling times within 1–2 s, compared to 5 s or more under LADRDC. These results validate the fixed-time design and demonstrate improved anti-disturbance capability and tracking accuracy.
3. The proposed controller structure is compact, requires moderate parameter tuning, and is compatible with real-time industrial PLC deployment. These properties make FT-ADRCDC a promising solution for high-speed, high-accuracy intake environment control in high-altitude test facilities. Future work will investigate its scalability to more complex multivariable test systems and its robustness under actuator constraints and sensor noise.

## Figures and Tables

**Table 1:** Control parameter settings.

Method	Core Controller	ESO
FT-ADRCDC	$\begin{array}{c} \alpha_{1}^{p}=0.6,\;\alpha_{2}^{p}=1.2,\\ \gamma_{1}^{p}=0.6,\;\gamma_{2}^{p}=1.2,\\ m_{1}^{p}=15,\;n_{1}^{p}=11,\\ \delta_{1}^{p}=3,\;q_{1}^{p}=7,\\ m_{2}^{p}=11,\;n_{2}^{p}=7,\\ \delta_{2}^{p}=3,\;q_{2}^{p}=7,\\ b^{p}=1,\\ \alpha_{1}^{\mathrm{Te}}=0.6,\;\alpha_{2}^{\mathrm{Te}}=1.2,\\ \gamma_{1}^{\mathrm{Te}}=0.6,\;\gamma_{2}^{\mathrm{Te}}=1.2,\\ m_{1}^{\mathrm{Te}}=15,\;n_{1}^{\mathrm{Te}}=11,\\ \delta_{1}^{\mathrm{Te}}=3,\;q_{1}^{\mathrm{Te}}=7,\\ m_{2}^{\mathrm{Te}}=11,\;n_{2}^{\mathrm{Te}}=7,\\ \delta_{2}^{\mathrm{Te}}=3,\;q_{2}^{\mathrm{Te}}=7,\\ b^{\mathrm{Te}}=1,\\ h=1.0 \end{array}$	$\begin{array}{c} \rho^{p}=0.8,\;\beta_{1}^{p}=0.9,\;\beta_{2}^{p}=1.1,\\ k_{1}^{p}=2,\;k_{2}^{p}=5,\\ l_{1}^{p}=18,\;l_{2}^{p}=108,\;l_{3}^{p}=216,\\ \theta^{p}=1,\\ \rho^{\mathrm{Te}}=0.8,\;\beta_{1}^{\mathrm{Te}}=0.9,\;\beta_{2}^{\mathrm{Te}}=1.1,\\ k_{1}^{\mathrm{Te}}=2,\;k_{2}^{\mathrm{Te}}=5,\\ l_{1}^{\mathrm{Te}}=18,\;l_{2}^{\mathrm{Te}}=108,\;l_{3}^{\mathrm{Te}}=216,\\ \theta^{\mathrm{Te}}=1 \end{array}$
LADRDC	$\begin{array}{c} {\mathrm{Prop}}_{1}=4,\;{\mathrm{Deri}}_{1}=8,\\ {\mathrm{Prop}}_{2}=4,\;{\mathrm{Deri}}_{2}=8 \end{array}$	$\omega^{p}=7,\;\omega^{Te}=7$

**Table 2:** Tracking performance comparison during t=0–180 s.

Category	Metric	FT-ADRCDC	LADRDC	Unit
Pressure tracking	MAR	0.899	1.271	kPa
	MSE	0.0821	0.1521	kPa^2^
	AIE	5.122	18.206	kPa·s
Temperature tracking	MAR	1.137	1.655	°C
	MSE	0.267	0.456	°C^2^
	AIE	5.013	22.689	°C·s

**Table 3:** Tracking performance comparison during t=110–180 s.

Category	Metric	FT-ADRCDC	LADRDC	Unit
Pressure tracking	MAR	0.899	1.271	kPa
	MSE	0.109	0.213	kPa^2^
	AIE	2.965	10.015	kPa·s
Temperature tracking	MAR	1.137	1.655	°C
	MSE	0.497	0.666	°C^2^
	AIE	3.851	13.332	°C·s

**Table 4:** Tracking performance comparison during t=110–180 s.

Category	Metric	FT-ADRCDC	LADRDC	Unit
Pressure tracking	MAR	0.372	0.843	kPa
	MSE	0.005	0.051	kPa^2^
	AIE	2.019	6.381	kPa·s
Temperature tracking	MAR	0.489	1.203	°C
	MSE	0.009	0.101	°C^2^
	AIE	2.625	8.435	°C·s

## Data Availability

The original contributions presented in this study are included in the article. Further inquiries can be directed to the corresponding author.

## Appendix A. Eight-Coefficient Polynomials for Dry Air

**Table A1:** Polynomial coefficients $A_{0}$–$A_{8}$ for $c_{p}\left(T\right)/R$ and h(T)/RT (valid for 199–2201 K).

$A_{0}$	$A_{1}$	$A_{2}$	$A_{3}$	$A_{4}$	$A_{5}$	$A_{6}$	$A_{7}$	$A_{8}$
0.992313	0.236688	−1.852148	6.083152	−8.893933	7.097112	−3.234725	0.794571	−0.081873

## References

[1] Hou M.J. (2014). High-Altitude Simulation Testing Technology.

[2] Davis M., Montgomery P. (2005). A flight-simulation vision for aeropropulsion altitude ground test facilities. J. Eng. Gas Turbines Power.

[3] Walker S., Tang M., Mamplata C. TBCC propulsion for a Mach 6 hypersonic airplane. Proceedings of the 16th AIAA/DLR/DGLR International Space Planes and Hypersonic Systems and Technologies Conference.

[4] Pei X., Wang X., Liu J., Zhu M., Dan Z., He A., Miao K., Xu Z. (2023). A review of modeling, simulation and control technologies of altitude ground test facilities for control application. Chin. J. Aeronaut..

[5] Lu L., Tian S., Xue D., Zhang T., Chen Y., Zhang S. (2019). A review of industrial MIMO decoupling control. Int. J. Control Autom. Syst..

[6] Guan J.C., Ren H.W., Tan G.L. (2024). Distributed dynamic event-triggered control to leader-following consensus of nonlinear multi-agent systems with directed graphs. Entropy.

[7] Zhao B., Wang D., Shi G., Liu D., Li Y. (2018). Decentralized control for large-scale nonlinear systems with unknown mismatched interconnections via policy iteration. IEEE Trans. Syst. Man Cybern. Syst..

[8] Garrido J., Vázquez F., Morilla F. (2014). Inverted-decoupling internal-model control for square stable multivariable time-delay systems. J. Process Control.

[9] Chekari T., Mansouri R., Bettayeb M. (2018). IMC–PID fractional-order filter multi-loop controller design for multivariable systems based on a two-degree-of-freedom control scheme. Int. J. Control Autom. Syst..

[10] Gouta H., Saïd S.H., Barhoumi N., M’Sahli F. (2017). Generalized predictive control for a coupled four-tank MIMO system using a continuous–discrete-time observer. ISA Trans..

[11] Noshadi A., Shi J., Lee W.S., Shi P., Kalam A. (2016). System identification and robust control of multi-input multi-output active-magnetic-bearing systems. IEEE Trans. Control Syst. Technol..

[12] Huang X., Song Y. (2023). Distributed and performance-guaranteed robust control for uncertain MIMO nonlinear systems with controllability relaxation. IEEE Trans. Autom. Control.

[13] Wang H., Yang C., Liu X., Zhou L. (2021). Neural-network-based adaptive control of uncertain MIMO singularly perturbed systems with full-state constraints. IEEE Trans. Neural Netw. Learn. Syst..

[14] Garrard D., Vaughn D., Milhoan A., Williams S., Choi S. Checkout testing of the new basic process-control system at the aerodynamic and propulsion test unit. Proceedings of the 18th AIAA/3AF International Space Planes and Hypersonic Systems and Technologies Conference.

[15] Garrard G.D. Hypersonic test capabilities at AEDC’s aerodynamic and propulsion test unit. Proceedings of the 53rd AIAA Aerospace Sciences Meeting.

[16] Bierkamp J., Köcke S., Staudacher S., Fiola R. Influence of ATF dynamics and controls on jet-engine performance. Proceedings of the ASME Turbo Expo 2007: Power for Land, Sea and Air.

[17] Weisser M., Bolk S., Staudacher S. (2013). Hardware-in-the-Loop Simulation of a Feedforward Multivariable Controller for the Altitude Test Facility at the University of Stuttgart.

[18] Dan Z., Zhang S., Bai K., Qian Q., Pei X., Wang X. (2021). Air-intake environment simulation of an altitude-test facility control based on an extended-state observer. J. Propuls. Technol..

[19] Lun Y., Wang H., Zhang S., Dan Z., Qian Q., Pei X., Yang Z. Continuous-state control of a flight-environment testbed based on state feedback and LPV. Proceedings of the International Conference on Autonomous Unmanned Systems.

[20] Zhu M., Wang X., Yang S., Chen H., Miao K., Gu N. Two-degree-of-freedom *μ*-synthesis control with a Kalman filter for flight-environment simulation volume with sensor uncertainty. Proceedings of the ASME Turbo Expo 2019: Power for Land, Sea and Air.

[21] Han J. (2002). From PID technique to active-disturbance-rejection control technique. Control Eng. China.

[22] Han J. (2008). Active-disturbance-rejection control technique—The technique for estimating and compensating the uncertainties. Active Disturbance-Rejection Control Technique.

[23] Gao Z. Scaling and bandwidth-parameterisation-based controller tuning. Proceedings of the American Control Conference.

[24] Zhang Z., Cheng J., Guo Y. (2021). PD-based optimal ADRC with an improved linear extended-state observer. Entropy.

[25] Chai J., Wang M., Li Z., Zhao X. (2023). Improved super-twisting sliding-mode control for single-phase T-type three-level converters based on a fixed-time extended-state observer. IEEE Trans. Transp. Electrific..

[26] Cui L., Jin N., Chang S., Zuo Z., Zhao Z. (2022). Fixed-time ESO-based fixed-time integral terminal sliding-mode controller design for a missile. ISA Trans..

[27] Chang S., Wang Y., Zuo Z. (2021). Fixed-time active-disturbance-rejection control and its application to wheeled mobile robots. IEEE Trans. Syst. Man Cybern. Syst..

[28] Boylston B.M. (2011). Quasi-One-Dimensional Flow for Use in Real-Time Facility Simulations. Ph.D. Thesis.

[29] McBride B.J., Zehe M.J., Gordon S. NASA Glenn Coefficients for Calculating Thermodynamic Properties of Individual Species. NASA TP-2002-211556, 1 September 2002. https://ntrs.nasa.gov/citations/20020085330.

[30] Bhat S.P., Bernstein D.S. (2000). Finite-time stability of continuous autonomous systems. SIAM J. Control Optim..

[31] Polyakov A. (2012). Nonlinear feedback design for fixed-time stabilization of linear control systems. IEEE Trans. Autom. Control.

